# Adhesion patterning by a novel air-lock technique enables localization and *in-situ* real-time imaging of reprogramming events in one-to-one electrofused hybrids

**DOI:** 10.1063/1.4965422

**Published:** 2016-10-27

**Authors:** S. Sakamoto, K. O. Okeyo, S. Yamazaki, O. Kurosawa, H. Oana, H. Kotera, M. Washizu

**Affiliations:** 1 Department of Bioengineering, School of Engineering, The University of Tokyo, Tokyo 113-3656, Japan; 2 Department of Mechanical Engineering, School of Engineering, The University of Tokyo, Tokyo 113-3656, Japan; 3 Center for Stem Cell Therapy, The Institute of Medical Science, The University of Tokyo, Tokyo 113-3656, Japan; 4 Department of Microengineering, School of Engineering, Kyoto University, Kyoto 606-8501, Japan

## Abstract

Although fusion of somatic cells with embryonic stem (ES) cells has been shown to induce reprogramming, single-cell level details of the transitory phenotypic changes that occur during fusion-based reprogramming are still lacking. Our group previously reported on the technique of one-to-one electrofusion via micro-slits in a microfluidic platform. In this study, we focused on developing a novel air-lock patterning technique for creating localized adhesion zones around the micro-slits for cell localization and real-time imaging of post fusion events with a single-cell resolution. Mouse embryonic fibroblasts (MEF) were fused individually with mouse ES cells using a polydimethylsiloxane (PDMS) fusion chip consisting of two feeder channels with a separating wall containing an array of micro-slits (slit width ∼3 *μ*m) at a regular spacing. ES cells and MEFs were introduced separately into the channels, juxtaposed on the micro-slits by dielectrophoresis and fused one-to-one by a pulse voltage. To localize fused cells for on-chip culture and time-lapse microscopy, we implemented a two-step approach of air-lock bovine serum albumin patterning and Matrigel coating to create localized adhesion areas around the micro-slits. As a result of time-lapse imaging, we could determine that cell division occurs within 24 h after fusion, much earlier than the 2–3 days reported by earlier studies. Remarkably, Oct4-GFP (Green Fluorescent Protein) was confirmed after 25 h of fusion and thereafter stably expressed by daughter cells of fused cells. Thus, integrated into our high-yield electrofusion platform, the technique of air-lock assisted adhesion patterning enables a single-cell level tracking of fused cells to highlight cell-level dynamics during fusion-based reprogramming.

## I. INTRODUCTION

Pluripotent stem cells produced by reprogramming somatic cells are increasingly attracting attention due to their potential application to stem cell therapy. Fusion with embryonic stem (ES) cells has been shown to induce reprogramming of somatic cells. Tada *et al*. were the first to report that the expression of endogenous Oct4-GFP (Green Fluorescent Protein) reporter in mouse embryonic fibroblasts (MEFs) could be triggered by fusion with ES cells within 48 h after fusion, and that the reprogrammed somatic cells had the potential to differentiate into the three germ layers when injected into a blastocyst.^1^ Subsequent studies have confirmed the suitability of somatic-ES cell fusion as an alternative strategy to reprogramming toward pluripotency.^2,3^

For a better understanding of the reprogramming process, a major point of interest lies in acquisition of detailed epigenetic and phenotypic information of fused somatic cells during the transitory process to pluripotency. Most researches have, however, focused on genetic and epigenetic characterization of the reprogramming process using techniques such as polymerase chain reaction with reverse transcription (RT-PCR) to carry out detailed molecular analysis of gene expression in multiple somatic cell hybrids sorted at specific time intervals after fusion by fluorescence-activated cell sorting (FACS). For instance, Bhutani *et al*. demonstrated that reprogramming requires activation-induced cytidine deaminase (AID)-mediated DNA demethylation.^2^ In addition, Tsubouchi *et al*. used RT-PCR to demonstrate that reprogramming efficiency improves when somatic cells are fused with ES cells in S/G2 phase, and went on to suggest that induction of reprogramming requires DNA synthesis.^4^

Although such molecular-level studies have contributed to the elucidation of molecular players involved in the reprogramming process, they have failed to capture the transitory phenotypic changes that occur during the process. In other words, information such as cell cycle dynamics and morphological changes that accompany reprogramming can only be gathered by continuous physical observation of individual cells right from the time of fusion. Such data will supplement results of molecular analyses and aid in gaining deeper insights into the reprogramming process, for instance, why only a few cells become fully reprogrammed.

Conventionally, fusion has been achieved using polyethylene glycol (PEG) or virus-based cell fusion.^5–7^ Although simple to implement, in particular, the popularly used PEG fusion method inflicts a significant cell damage, which can negatively impact the reprogramming process.^6,8^ In contrast, electrofusion offers several advantages, including minimal cell damage, high adaptability to different cell types, and simplicity of process control and implementation.^9–11^ Various strategies for cell manipulation and electrofusion in a microfluidic platform have been reported.^12–14^ Notably, our group previously developed the technique of one-to-one electrofusion via micro-orifices (slits) that employs electric field constriction at micro-orifices to achieve both cell alignment by dielectrophoresis (DEP), and subsequently, cell fusion in a microfluidic platform.^15,16^ The technique overcomes the limitation of cell size difference, and damage to cells is extremely reduced since membrane breakdown and fusion occur only at the point of cell-cell contact within an orifice (ϕ = 2–3 *μ*m).^17^

To achieve on-chip culture and seamless tracking of the hybrids, we developed and implemented a novel air-lock patterning technique to create adhesion zones on the channel floor around micro-slits where fused cells were localized for time-lapse imaging. Here we demonstrate that this approach enables adhesion patterning of Matrigel for localization of fused cells, thereby permitting extended *in-situ* time-lapse imaging to monitor post-fusion reprogramming events. In addition, since the rest of the channel regions are bovine serum albumin (BSA)-coated, unfused cells can be flushed to avoid interfering with imaging. Experimental results involving one-to-one fusion of Oct4-GFP MEFs with ES cells revealed that cell-division and the onset of Oct4 expression occur in about 24 h after fusion, much faster than the 2–3 days reported by earlier studies.^2^

## II. METHODS

### A. Cell culture

Mouse ES cells (B6 cell line) were cultured in ESGRO medium (Millipore, Germany) containing leukemia inhibitory factor (LIF) and bone morphogenetic protein 4 (BMP4). The medium was supplemented with glycogen synthase kinase 3β inhibitor (GSK3βi) supplement, which is necessary for maintaining pluripotency of ES cells.^18^

For somatic cells, we used mouse embryonic fibroblast MEFs containing an endogenous Oct4-GFP reporter that fluoresces green, when reprogramming to pluripotency is successfully initiated after fusion. MEFs were cultured in Dulbecco's Modified Eagle Medium (DMEM)/F12 supplemented with 10% fetal bovine serum (FBS).

Fused cells were cultured in ESGRO medium to avoid differentiation of ES nuclei. However, because ESGRO has low nutrients, it was supplemented with 1% FBS to support the survival of MEFs. GSK3βI was not added to the medium.

### B. High-yield one-to-one fusion using a PDMS microfluidic device

**FIG. 1.**
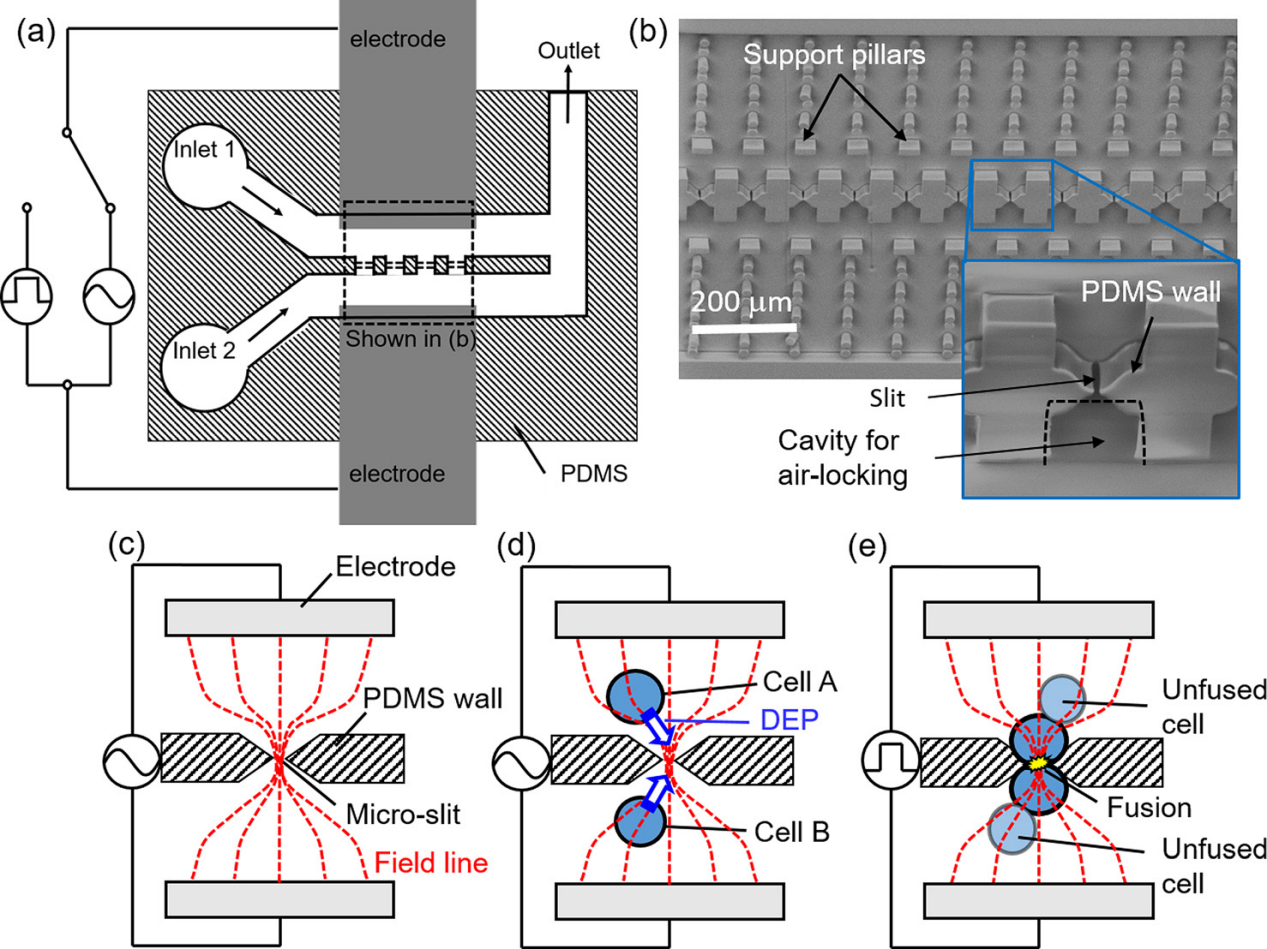
Configuration of PDMS fusion device and principle of one-to-one fusion. (a) A schematic of fusion device and circuitry. (b) An actual SEM image showing features on the PDMS device at the region boxed in (a). Inset in (b) is a magnified image showing a micro-slit for fusion and a micro-cavity for air-lock assisted BSA patterning. (c)–(e) Schematic representations of one-to-one fusion via micro-slits employing electric field constriction.

In this study, we employed the technique of one-to-one electrofusion via micro-orifices or micro-slits previously reported by our group.^15,16^ The microfluidic PDMS device used for fusion was fabricated by photolithography. It consisted of two parallel feeder channels separated by a vertical PDMS wall with micro-slits (slit width 3–4 *μ*m) for cell alignment by DEP and fusion by pulsation. The device was bonded onto a polystyrene culture dish which was patterned beforehand with aluminum electrodes at a spacing of 400 *μ*m. During bonding, the PDMS device was aligned such that the micro-slits come in between the aluminum electrodes, as shown in Fig. 1(a). A representative scanning electron microscope (SEM) image of a PDMS fusion device with an array of 50 slits at an interval of 100 *μ*m is shown in Fig. 1(b). Micro-cavities (marked in the inset in Fig. 1(b)) were created around the micro-slits to serve as pockets for air-lock assisted BSA patterning, which is explained in detail in Section II C.

For visualization of the fusion process, ES cells and MEFs were each labelled with 1 *μ*g/ml calcein AM and 1 *μ*g/ml calcein red orange for 2 min, centrifuged and then resuspended in a low-conductivity (100 *μ*S/cm) fusion buffer (100 mM sorbitol, 0.1 mM calcium acetate, 0.5 mM magnesium acetate). Next, labelled cells were loaded separately into each inlet port (shown in Fig. 1(a)) and then introduced into the channels by sucking buffer from the outlet port. For cell alignment, a high-frequency alternating current (1.0 MHz, 10V_p-p_) was applied to attract cells to the micro-slits by DEP induced by electric field constriction at the micro-slits (Figs. 1(c) and 1(d)). After confirming cell pair formation at all micro-slits, a pulse voltage (100 *μ*s, 10V_p_) was applied to initiate fusion (Fig. 1(e)). The success of fusion was monitored by dye mixing between fused cell pairs. Importantly, only cells in contact at the micro-slits take part in fusion which occurs in a one-to-one fashion.

### C. Air-lock patterning of cell adhesion areas for fused cells localization

**FIG. 2.**
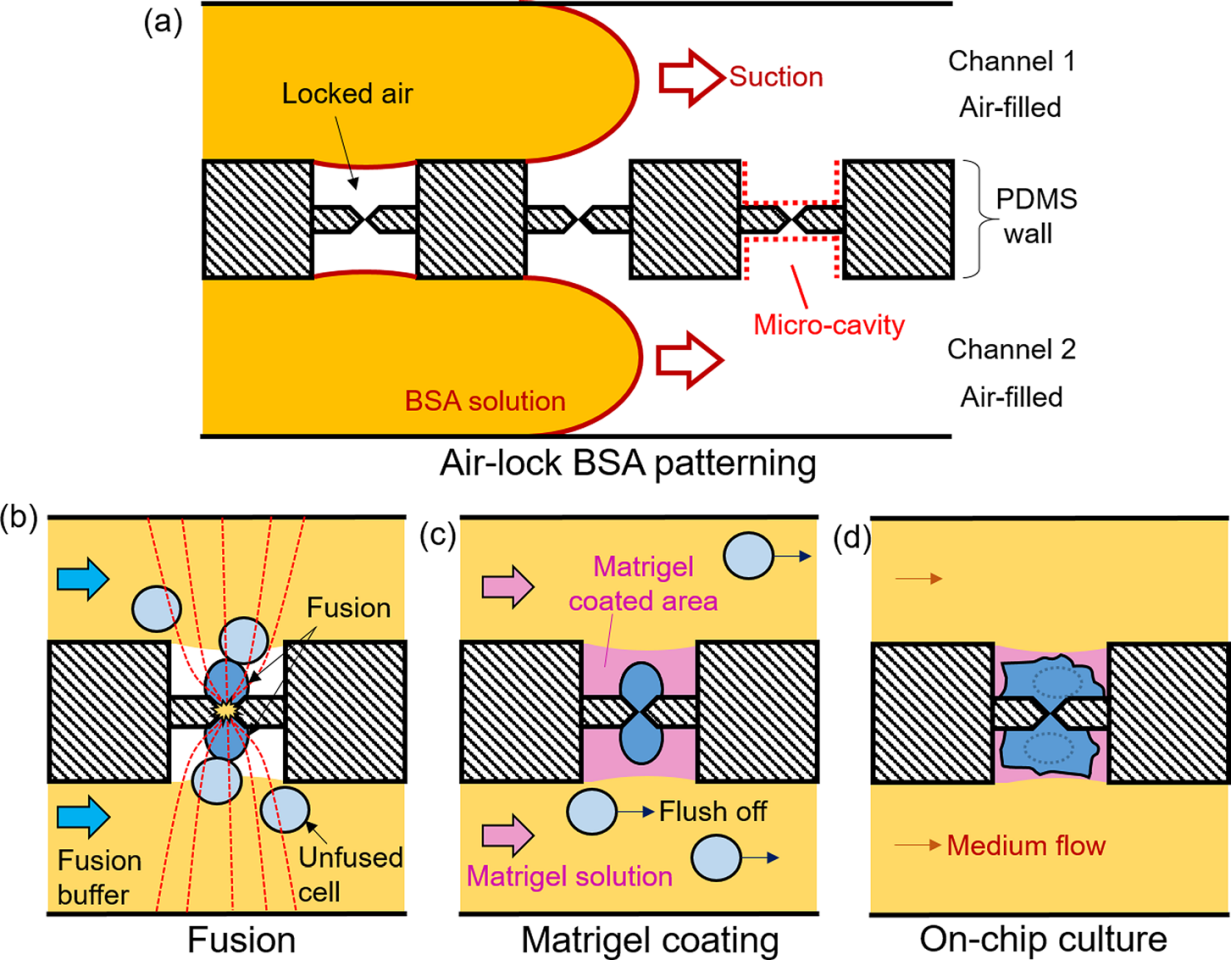
Procedures for creating localized adhesion zones for cell localization. (a) Air-lock assisted BSA patterning, (b) One-to-one electrofusion after BSA patterning, (c) Matrigel perfusion to coat BSA-free micro-cavities, (d) On-chip culture and subsequent imaging of fused cells.

To localize fused cells for extended microscopic imaging, we implemented a two-step patterning approach to create localized adhesion areas around the micro-slits (Fig. 2). In the first step, 0.1% BSA solution (in water) was perfused into the channels by suction as shown in Fig. 2(a), followed by incubation for 15 min at room temperature. During this process, air locked in the micro-cavities (indicated by red dotted lines in Fig. 2(a)) around the micro-slits prevents the penetration of BSA solution into the cavities, which therefore remain BSA-free. We call this “air-lock assisted BSA patterning” or simply “air-lock BSA patterning.” Other than the micro-cavities, the rest of the channel floor becomes BSA-coated and therefore resistant to cell adhesion. After BSA patterning, the device was vacuumed before cells were introduced and fused as illustrated in Fig. 2(b). We chose BSA because it is biocompatible and its property to block nonspecific protein adsorption onto PDMS is well-established.^19^

In the second step, Matrigel solution (10 *μ*g/ml in culture medium) was perfused to coat BSA-free micro-cavities (Fig. 2(c)), making these area adhesion competent. Matrigel is a widely used extracellular matrix (ECM) for stem cell culture. Since BSA resists protein adsorption,^19^ the matrix protein is excluded from the BSA-coated channel floor but instead become adsorbed onto the BSA-free areas. In this way, we could create localized Matrigel-coated adhesion areas around the micro-slits onto which fused cells could successfully adhere (Fig. 2(d)), enabling microscopic imaging for an extended period of time (more than 5 days). It should be noted that unfused cells (Fig. 2(c), light blue cells) were purged off by medium flow to avoid interference with imaging.

### D. On-chip culture and Imaging

After fusion, on-chip culture and imaging was performed. For medium exchange, the whole chip was completely immersed in a culture medium contained in a 60 mm culture dish. The device channels were perfused continuously with fresh medium from a reservoir located upstream of the channels. Flow was controlled by adjusting the height difference (pressure head) between the reservoir and the channel. The whole medium was replaced after every 3 days. Time-lapse microscopy was performed using Biorevo BZ-9000 all-in-one microscope (Keyence, Japan) fitted with an incubation chamber set at 37 °C and 5% CO_2_.

Other images were captured using Olympus IX71 (Olympus, Japan) fitted with Watec 2215 camera (Watec, Japan). Images were slightly processed for presentation using Image J (NIH, USA).

## III. RESULTS

### A. Result of air-lock patterning for the creation of localized adhesion areas

**FIG. 3.**
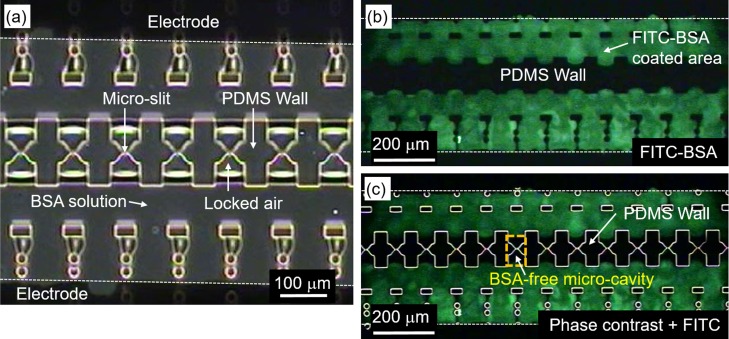
Result of BSA patterning. (a) Phase contrast image showing air locked inside the micro-cavities after perfusion of BSA solution. BSA penetration is excluded from the micro-cavities. (b) Visualization of air-lock assisted BSA patterning using FITC labelled BSA solution. (c) Merged image of phase contrast and FITC-BSA fluorescent images to illustrate successful air-lock BSA patterning.

For extended imaging, it was necessary to create localized adhesion zones where cells could be cultured on-chip. To achieve this, we performed an air-lock BSA patterning by first perfusing the channels of the PDMS fusion chip with 0.1% BSA solution without vacuuming the channels. Figure 3 shows the distribution of BSA on the channel floor of the PDMS chip after perfusion. As shown in Fig. 3(a), the micro-cavities around the micro-slits contain locked air (marked “locked air”), which occurs as BSA flows into the parallel air-filled channels. We refer to this phenomenon as “air-locking.”

To better visualize the effect of air-locking on BSA patterning, we used BSA conjugated with the green FITC fluorophore (FITC-BSA). The result shown in Fig. 3(b) clearly shows that most regions of the channel are covered by the green FITC-BSA. Fig. 3(c), which is a composite image of the bright field image in Fig. 3(a) with the fluorescence image in Fig. 3(b), further illustrates the coverage of BSA with respect to the micro-slits on the PDMS wall. It is clear from the figure that air-locking effectively prevents penetration of BSA into the micro-cavities around the micro-slits, which therefore remain unlabeled and appear dark as in Fig. 3(c). Thus, the region around the micro-slits remains BSA-free even as the rest of the chip floor becomes blocked by BSA to prevent random cell attachment during cell loading and culture. It should be noted that air-lock BSA patterning was performed prior to fusion.

After air-lock BSA patterning, excess BSA was removed and the channels vacuumed to enable cell loading, and, subsequently, fusion. Now, for stem cell adhesion, it was necessary to coat the BSA-free micro-cavities with Matrigel for subsequent on-chip cell culture. Matrigel is a widely used extracellular matrix which has been shown to support stem cell adhesion and growth. For Matrigel coating, we again perfused the channels with 10% Matrigel immediately after cell fusion, as illustrated in Fig. 2(b).

It should be stressed that Matrigel coating was an important step of the patterning process that enabled us to turn BSA-free micro-cavities into areas competent for cell adhesion and culture of stem cells. Thus, at the end of the procedures outlined above, the fusion chip now consisted of BSA-blocked channels and Matrigel-coated micro-cavities where cells were localized for extended imaging after fusion. Since Matrigel perfusion is done after fusion, it is necessary to carefully control the flow rate to avoid flushing off fused cells trapped at the micro-slits (our flow rate was around 10 *μ*l/h).

### B. One-to-one electrofusion

**FIG. 4.**
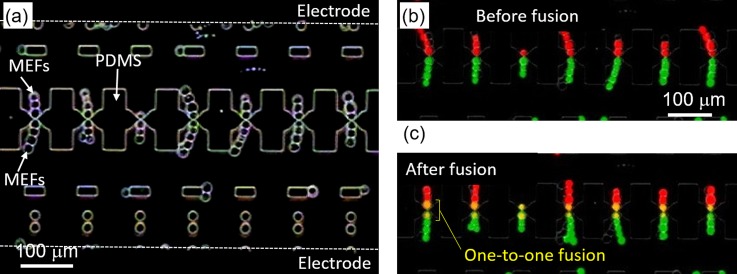
Results of one-to-one cell fusion. (a) Cells form a pearl chain at micro-slits due to electric field effect. (b) A fluorescent image of calcein-labelled MEFs forming a pearl chain at micro-slits before fusion. (c) Dye mixing between fused cells at micro-slits after fusion.

To demonstrate the capability of our electrofusion method to generate individually fused cells, in this experiment we fused two sets of MEFs labeled with either calcein red-orange AM (red fluorescence) and calcein AM (green fluorescence). Figure 4(a) shows MEFs aligned by DEP on either side of the micro-slits on a separating PDMS wall. Subsequently attracted cells formed pearl chains due to electric field effect. It should be noted that excess cells in the flow channel were purged off after successful alignment by DEP, leaving only cells forming pearl chains within the micro-cavities (Fig. 4(a)).

Images in Figs. 4(b) and 4(c) are composite images of the red and green fluorescence images corresponding to the labeling dyes. Since the micro-slits are only ∼3 *μ*m in width, cells cannot pass through, hence the red and green cells remain clearly separated (Fig. 4(b)). Upon application of a pulse voltage, fusion was initiated between cells directly in contact via the micro-slits, resulting in dye mixing by diffusion. The fused cells now appear yellow in the merged image in Fig. 4(c). Remarkably, the length of dye mixing corresponds to the diameter of a single cell, clearly indicating that only cells in contact via the micro-slits participate in fusion. This is attributable to the fact that maximum potential drop occurs at the micro-slits due to electric field constriction, inducing membrane breakdown specifically at the point of contact of the two cells in contact at the micro-slit. Other cells in the pearl chain remain unaffected.

It can be noticed that all the 7 pairs shown in the representative image in Fig. 4(c) are successfully fused one-to-one. Based on the number of aligned versus fused cell pairs, we determined the fusion efficiency to be more than 90%, proving the capability of our device for high-yield one-to-one electrofusion. Overall, our one-to-one fusion via micro-slit technique achieves one-to-one fusion with high efficiency compared to conventional bulk electrofusion where cells in the pearl chain are randomly fused.^13^ In addition, since electric-field constriction at the micro-slits amplifies an effective voltage, fusion can be achieved at a low voltage of ∼10 V thus minimizing cell damage.

### C. Localization of fused cells for time-lapse imaging

**FIG. 5.**
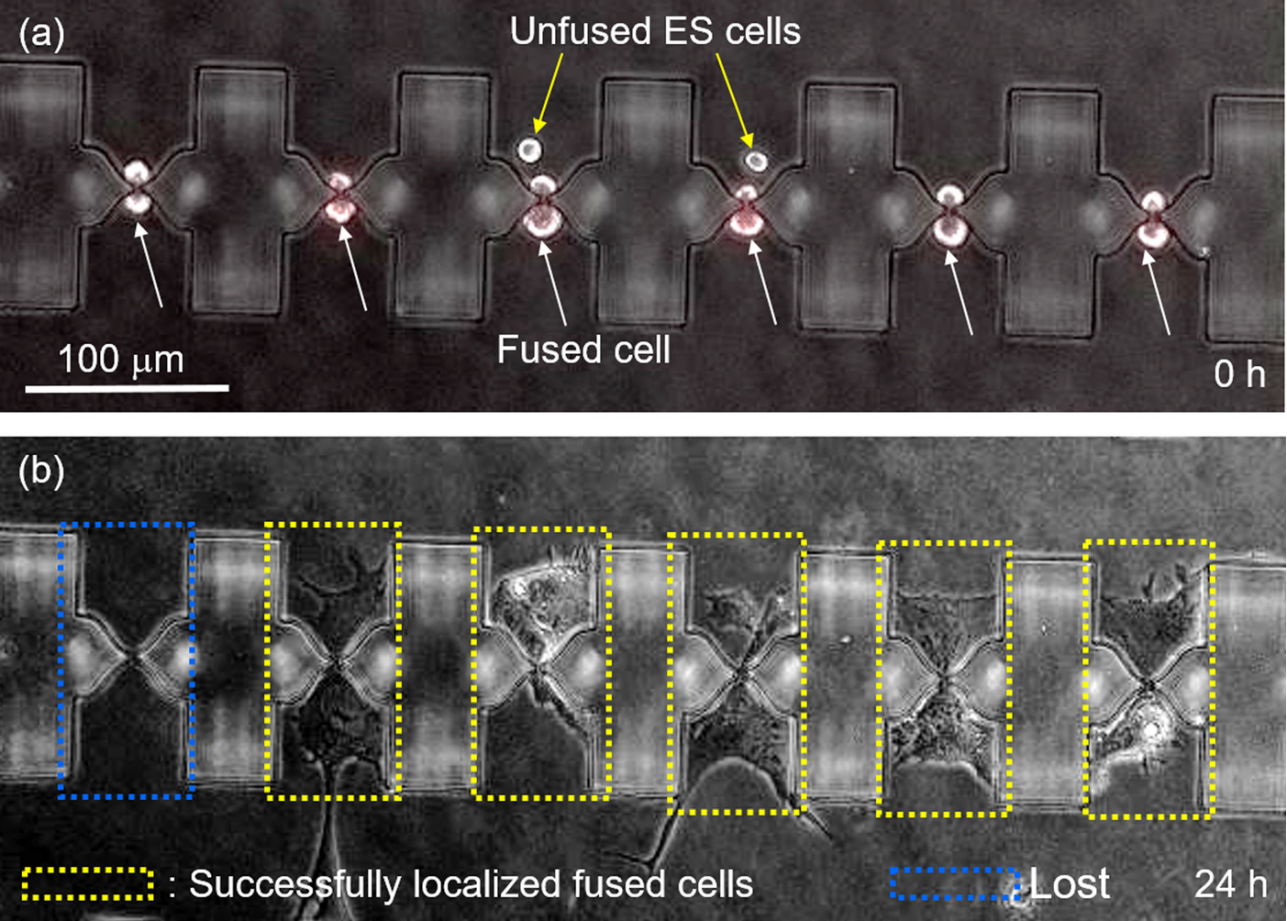
Result of localization of fused cells on adhesion zones for time-lapse imaging. (a) Fused cells aligned at micro-slits soon after fusion. (b) Fused cells adhered on Matrigel coated micro-cavities 24 h after fusion.

Figure 5 illustrates the capability of the adhesion zones created by air-lock BSA patterning and Matrigel coating to localize cells on the micro-cavities around the micro-slits, permitting *in-situ* imaging inside a microfluidic chamber. Soon after fusion, the six cell pairs shown in Fig. 5(a) are all expressing the red fluorescence, indicating a successful fusion. Two unfused ES-cells trapped inside the micro-cavities are also visible (Fig. 5(a), yellow arrows). At this time point, the hybrids are yet to adhere and appear round in shape. However, as shown in the supplementary material, Movie S2, these cells began to adhere onto the floor of the micro-cavities as early as 20 min after the start of on-chip culture under constant perfusion with fresh culture medium. Remarkably, cell extension occurred on either side of the micro-cavities and cells remained localized for the duration of imaging, which was in some cases over 5 days (Fig. 5(b)). Active cell division was also observed, with cells rounding up, dividing, and then reattaching to the adhesion zones (supplementary material, Movie S2). Remarkably, cell division was observed as early as 2 h after fusion, a strong indication of good cell viability. Thus, we argue that fusion across the micro-slits did not have a negative influence on cell viability.

It should be noted that the restriction imposed on cells by the micro-slits depends on the presence of the nucleus but not on the size of the cytoplasm, since the latter is highly flexible and can penetrate through even as the nuclei get trapped, especially after cell adhesion. This implies that cells can easily penetrate through the micro-slits during metaphase when the nuclear membrane breaks down. It is well known that cells in S-M phases of the cell cycle are relatively larger in size compared to those in other phases. Thus, it is not surprising that some cells that appear larger could penetrate through the micro-slits while apparently smaller ones become trapped, as rightfully pointed out by the reviewer.

Occasionally, some fused cells were lost during imaging after being swept off by the medium flow (blue dotted box in Fig. 5(b)). This occurred mostly during cell division when cells are briefly detached. Such cells would in some cases accumulate downstream of the feeder channels, and as mentioned later in Section III D, they could successfully form colonies. Additionally, imperfect BSA coverage of the channel floor resulted in some cells extending from the micro-cavities to the channel floor during on-chip culture (see Movie S2).

### D. On-chip culture and live imaging of Oct4-GFP expression

**FIG. 6.**
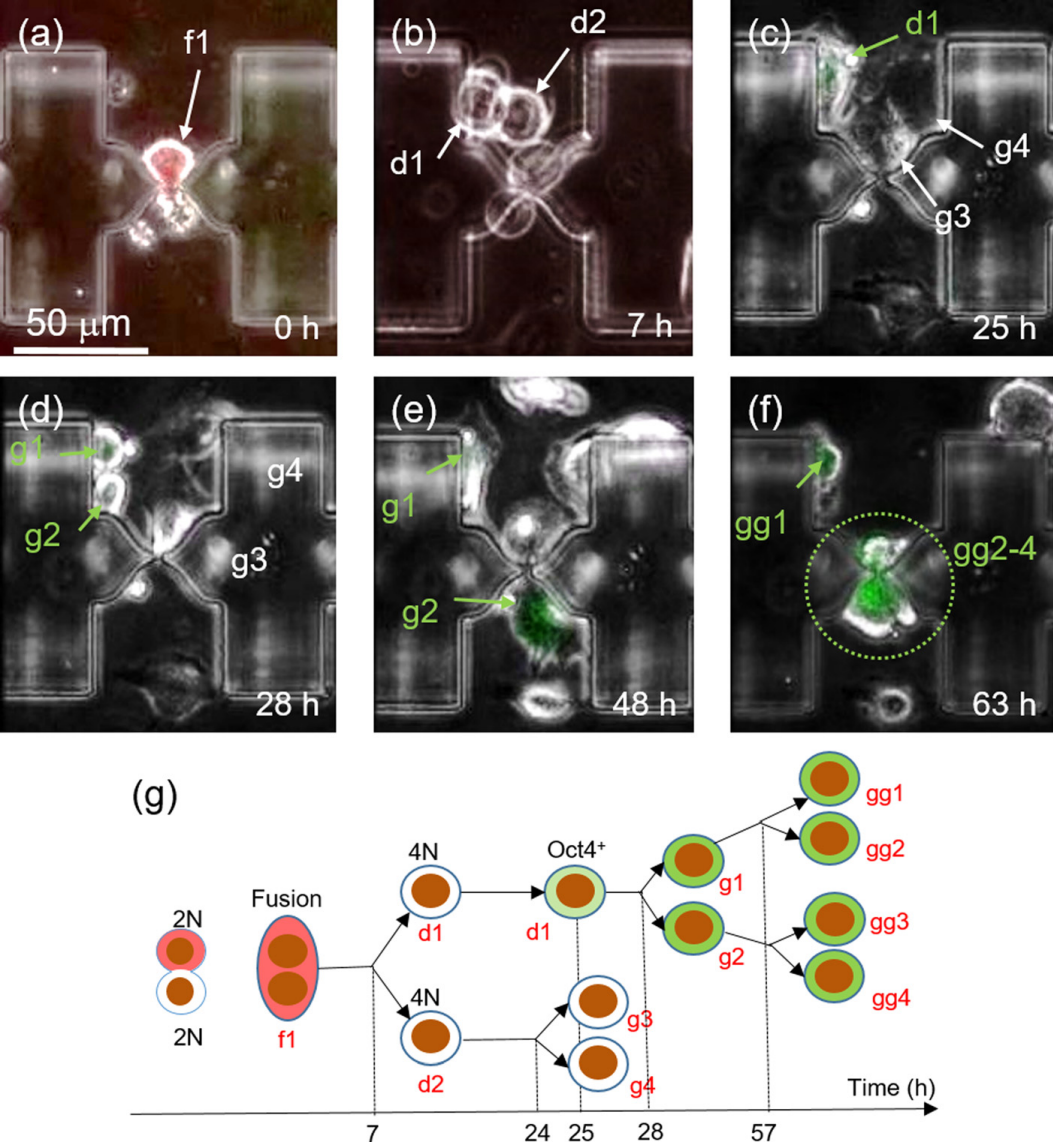
Result of post-fusion tracking of Oct4-GFP expression in fused cells. (a) A fused cell on a micro-slit at the start of time-lapse imaging. (b) The hybrid cell in (a) undergoes cell division after 7 h post fusion. (c) One of the daughter cell formed in the first cell division expresses the green Oct4-GFP fluorescence. (d) The cell marked “d1” in (c) divides into two granddaughter cells (g1, g2). (e) Both granddaughter cells express the green Oct4-GFP fluorescence 48 h post fusion. (f) Great granddaughter cells formed by cells in (e) express Oct4-GFP 63 h post fusion. (g) Tree diagram summarizing the time-transiting information of a fused cell resulting in green fluorescence.

Following successful one-to-one fusion, we performed time-lapse microscopy to monitor the behavior of fused cells on chip. Fig. 6 shows a representative ES-MEF hybrid whose dynamics was captured by time-lapse microscopy (also see Video S3, supplementary material). The fused cell (marked f1 in Fig. 6(a)) underwent the first cell division at 7 h after fusion, giving rise to two daughter cells (marked d1 and d2 in Fig. 6(b)). Remarkably, the daughter cell, marked d1, began to display slightly the green fluorescence of OCT4-GFP at 25 h after fusion (Fig. 6(c)), suggesting the onset of reprogramming as reported earlier. The same cell underwent the second division at 28 h after fusion (Fig. 6(c)), and thereafter, showed an increase in the level of GFP expression with time. Indeed, the green fluorescence was markedly more visible at 48 hours after fusion (Fig. 6(e)), possibly due to an increase in accumulated GFP levels. The third division of granddaughter cells occurred at 60 h after fusion. It should be stressed that successful localization of fused cells and their progenies on patterned adhesion areas around micro-cavities enabled us to trace Oct4-GFP continuously until all GFP-positive cells died after 65 h following fusion, probably due to over excitation (Fig. 6(f)).

**FIG. 7.**
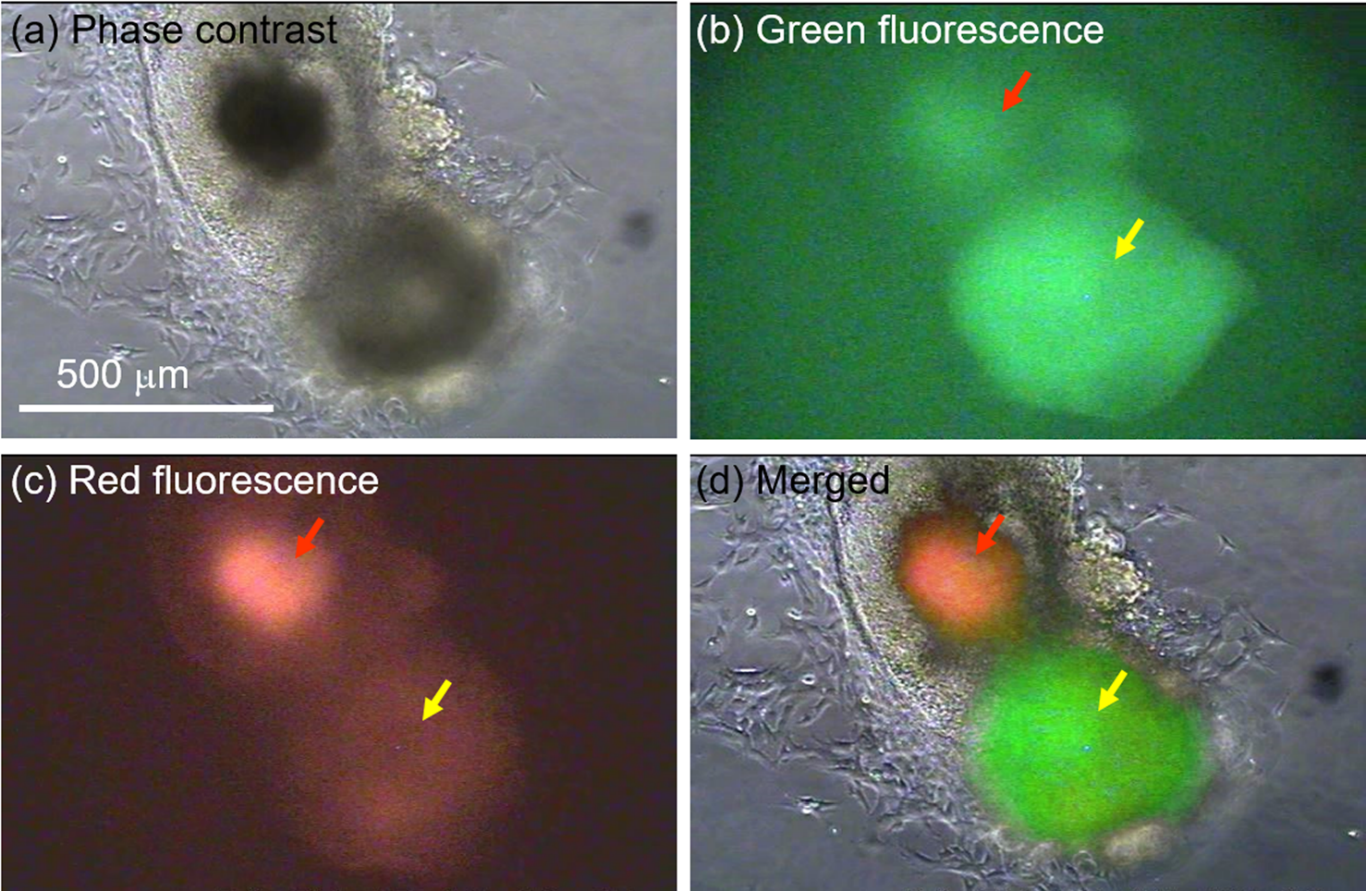
GFP positive cell colony formed on chip after two weeks of continuous culture inside an incubator. (a) Phase contrast image showing colonies of cells formed downstream of feeder channels. (b). Fluorescence imaging reveals a relatively strong expression of the green GFP fluorescence by one of the two neighboring colonies. (c) Fluorescence imaging to discriminate between autofluorescence and GFP expression. (d) A composite image illustrating the difference in expression between the two neighboring colonies. The greener one represents GFP expression while the reddish indicates autofluorescence.

The tree diagram in Fig. 6(g) summarizes the time course of cell division and Oct4 expression exhibited by the representative hybrid and its progeny. The most striking feature is the asymmetric expression of Oct4-GFP at 25 h after fusion, i.e., only one of the two daughter cells (marked “d1”) expressed the green fluorescence. The reason for this is not clear and remains to be investigated. Remarkably, the expression of Oct4-GFP was successfully confirmed in three subsequent generations, suggesting stability of expression and epigenetic transition toward pluripotency. Furthermore, colony formation by GFP-positive cells was confirmed in a separate experiment for cells that had accumulated downstream of the feeder channels. In this particular experiment, on-chip culture was done inside a humidified incubator (37 °C and 5% CO_2_), not on a microscope stage. After 2 weeks, we successfully obtained a sizable Oct4-GFP positive colony (500 *μ*m in diameter) at the periphery of another colony of unfused ES cells (Figs. 7(a) and 7(b)). The Oct4-GFP positive colony shows a markedly stronger expression of the green Oct4-GFP fluorescence compared with the red autofluorescence (Fig. 7(c)), unlike the ES cell colony with a high expression of autofluorescence attributable to cell death by necrosis. The clear difference between autofluorescence and GFP expression is clearly illustrated in Fig. 7(d), which is a composite of the images in Figs. 7(a)–7(c).

Overall, adhesion patterning enabled localization of hybrids for on-chip culture and extended imaging, revealing that cell division and reprogramming can occur within about 24 h after a successful one-to-one cell fusion. In addition, the formation of a GFP-positive colony on-chip by fused cells further alludes the stability of the Oct4-GFP expression and hints to the possibility of reprogramming. However, it should be pointed out that the present study focused on a proof-of concept, and therefore chose a simple chip configuration with empirically designed micro-cavities that could hold cells only for the duration of observation necessary to monitor GFP expression (about 5–6 days). In other words, the size of the Matrigel-coated zone could only accommodate a few cells and might have limited a further increase in cell number by contact inhibition. For longer-term cell culture and imaging on chip, it will be necessary to design chips with larger micro-cavities, and this remains the subject of our future study.

## IV. DISCUSSION

Observable physiological changes that occur after fusion of somatic cells with ES cells can be regarded as manifestations of the underlying genetic and epigenetic changes that accompany reprogramming toward pluripotency. This study has presented a microfluidic device that enables both one-to-one electrofusion of ES cells with MEFs as well as post-fusion real-time tracking of the resulting hybrids. The fusion yields 100% heterogeneous pairs. We have also presented a novel on-chip adhesion patterning and demonstrated its capability to achieve localization of fused cells for extended on-chip culture and imaging.

Although various studies have attempted fusion in a microfluidic system,^13,20,21^ subsequent on-chip culture and imaging has proved to be challenging and only attempted by a few studies.^12,22^ The challenges of continuous culture inside a PDMS device are well documented.^23^ This study overcomes these challenges and achieves localization of fused cells for culture and imaging on-chip with a single-cell resolution. For this purpose, we developed a two-step adhesion patterning approach of BSA patterning followed by Matrigel coating. Besides cell localization, this approach also enables excess unfused cells to be flushed off to avoid interference with imaging. Air-lock patterning could be achieved because BSA was introduced into the fusion device without vacuuming, i.e., the device was air-filled. Otherwise BSA penetration into the micro-cavities surrounding the slits would occur, rendering the process impossible. Apparently, the width and length (the distance from the channel edge to the slit) of a micro-cavity influence the effectiveness of air-lock assisted BSA patterning. In essence, air-locking can be more effective when the width is small and the length is large enough such that meniscus force is balanced off by the resistance of the trapped air. The width of the micro-cavity is, however, limited by the size of the cell to be fused. In our experiment, we determined these parameters empirically, aiming to achieve both air-locking as well as easy cell alignment on the micro-slits.

For the suppression of cell adhesion in the main channels, we chose BSA for surface modification via physical adsorption due to its tremendous simplicity and efficiency compared to other covalent surface modification methods. Indeed, BSA is the most conventionally used material for surface modification by physical adsorption because it is easy to prepare and implement, and it works with both glass and PDMS.^24^ Other than BSA, Poly(ethylene glycol) (PEG), also known as poly(ethylene oxide) (PEO), is a well-known material for preventing nonspecific adsorption of proteins,^24^ and has good properties of biocompatibility and low toxicity. In addition, PEO-derivatives such as pluronic and Poly(L-lysine)-graft-poly(ethylene glycol) (PLL-g-PEG) are also excellent passivating agents suitable for both glass and PDMS surfaces.^25^ For instance, stable cell patterning experiments have been demonstrated by Tan *et al*.^26^ and Liu *et al*.^27^ using Pluronic F108 on various surfaces, including glass, PDMS, and polystyrene.

The capability to localize cells on the adhesion zones (micro-cavities) enabled us to carry out time-lapse microscopy to determine the onset of cell division and GFP-expression. Both were confirmed to occur within 24 h after fusion, in contrast to 2–3 days reported for the case of PEG fusion.^2,4,28^ This can be attributed to the differences in the degree of cell damage between electric field constriction-based electrofusion method employed in our study, and PEG-induced fusion used by others. In our method, micro-slits induce electric field constriction and, in effect, membrane breakdown leading to fusion can be achieved at a low voltage with minimal cell damage.^15,17^ Membrane breakdown is limited to the point of contact between the cell pairs at the micro-slits,^17^ thus damage to the entire cell is minimal. In contrast, PEG method used in previous studies is known to cause considerable cell damage, which may interfere with cell cycle progression, and hence a delay in cell division.^6,8^

Considering Oct4-GFP expression as an indicator of reprogramming, the yield of our one-to-one fusion method was considerably low as only 1 out of 100 hybrids in each fusion experiment exhibited Oct4-GFP expression. In contrast, previous studies using RT-PCR detection method have shown that the efficiency of generating Oct4 positive cells can be as high as 70%.^2^ The low efficiency can be due to one-to-one fusion in which case the amount of reprogramming factors in a single ES cell may be insufficient to induce steady Oct4-GFP expression, i.e., reprogramming. However, comparison with other studies is limited by the fact that few studies have considered a fusion-based reprogramming for the case of one-to-one fusion.

Another plausible reason for the low efficiency may be the low sensitivity of our detection method, which relied solely on the expression of Oct4-GFP. In other words, since endogenous Oct4-GFP reporter only comes alive under sufficiently high protein expression levels, it is less sensitive and cannot capture cells with low expression levels that would otherwise be detected by highly sensitive methods such as RT-PCR. Indeed, we did determine by immunofluorescence that Oct4 is present in the nuclei of fused MEFs as early as 8 h after fusion (data not shown). Therefore, it is plausible to predict that the yield of our method, in terms of the number of fused somatic cells expressing Oct4 above normal base levels, would have been much higher if analyzed using more sensitive methods. Future studies will consider using RT-PCR to more accurately determine the expression of Oct4 in singly fused MEFs.

It should be noted that we consider the effect of microslits on cell viability and therefore GFP expression to be minimal. First, time-lapse movies (supplementary material Movie 2) show cells adhering on the floor of the microcavities as early as 20 min after fusion, and continues to move and divide actively during the entire observation period, a strong indication of good cell viability. Second, cells are not entirely restricted, but are able to pass through the microslits, as shown in Movie 2 (supplementary material). Moreover, although narrow in width, the height of the micro-slits in the orthogonal direction is nearly 30 *μ*m, implying that the height-wise restriction is minimal. Furthermore, fused cells adhered inside the micro-cavities are cushioned from the effect of shear stress due to the relatively rapid flow in the main channel. Thus, we can argue that fused cells inside the micro-cavities are exposed to negligibly low levels of mechanical stress whose effect on cell viability can be ignored, as illustrated by the fact that cells are able to maintain viability during the period of time-lapse imaging inside the micro-channels.

Overall, this study has demonstrated the capability of achieving real-time imaging of fusion-based reprogramming events in heterogeneous pairs obtained by a one-to-one electrofusion device integrated with adhesion zones created by a novel air-lock patterning approach. We have not only shown the possibility of single-cell level on-chip tacking of reprogramming events, but also demonstrated the possibility of achieving reprogramming by one-to-one fusion of somatic cells with ES cells. As a proof-of-concept study, we chose to work with a small chip with a simple configuration that could only accommodate up to 50 cells. However, the system can be scaled up to perform the fusion of a large number of cells simultaneously using an orifice plate (sheet) which contains thousands of arrayed micro-orifices, as previously reported by our group.^14^

## V. CONCLUSION

In this study, we performed an on-chip real-time tracking of somatic cells with ES cells to determine the phenotypic changes that precede reprogramming to pluripotency with a single-cell resolution. Fusion between MEFs and ES cells was successfully conducted using a high-yield one-to-one fusion with field constriction technique on a microfluidic platform. A novel adhesion patterning on the channel floor of the microfluidic device enabled us to localize fused cells for time-lapse microscopy. As a result, we observed cell division within 24 h after fusion, much earlier than previously reported. We also managed to capture the onset of Oct4-GFP expression in the ES-MEF hybrids, which occurred at 25 h after fusion. Thus, this study has demonstrated the possibility of single-cell level on-chip tracking of reprogramming events, and also demonstrated the possibility of achieving reprogramming by one-to-one fusion of MEF cells with ES cells.

## SUPPLEMENTARY MATERIAL

See supplementary material for three videos that have been provided to aid in the understanding of the study results. Video S1 shows cell manipulation inside a PDMS microfluidic device during loading, DEP alignment, and fusion. Video S2 shows cell localization on adhesion zones created by our novel air-lock BSA patterning and Matrigel coating. Finally, Video S3 shows time-lapse monitoring of a representative fused pair of MEF and ES cell from the time of fusion to the expression of Oct4-GFP by subsequent progenies.
